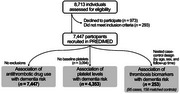# Plasma Platelet Concentration and Coagulation Biomarkers in Dementia and Alzheimer's Disease

**DOI:** 10.1002/alz70856_107222

**Published:** 2026-01-08

**Authors:** Olga Castaner, Mireia Malcampo, Isaac Subirana, Montserrat Fito, Emilio Ros

**Affiliations:** ^1^ CIBERESP, Madrid, Spain; ^2^ Hospital del Mar Research Institute, Barcelona, Spain; ^3^ CIBERCV, Madrid, Spain; ^4^ CIBEROBN, Madrid, Spain; ^5^ Institute of Biomedical Research August Pi i Sunyer, Hospital Clínic, Barcelona, Spain

## Abstract

**Background:**

Dysregulation of platelet function and coagulation has been implicated in Alzheimer's disease (AD) pathogenesis. Increased platelet activation is linked to vascular inflammation and cognitive decline. Elevated coagulation factors (fibrinogen, *p*‐selectin) exacerbate neurovascular dysfunction, while Factor V has shown a potential neuroprotective role. These findings suggest that platelet and coagulation biomarkers may serve as early indicators of dementia. Our objective is to assess the associations between plasma platelet concentration, anticoagulant drug use, and coagulation factors, with dementia risk.

**Method:**

This study analyzed PREDIMED cohort data, a Spanish multicenter clinical trial on cardiovascular risk. Two study designs were used: 1) Sub‐cohort analysis (7,447 participants) to assess platelet levels, antiplatelet drug use with dementia risk; 2) Nested Case‐Control study (95 dementia cases, 158 controls) evaluating the association of thrombosis biomarkers (fibrinogen, *p*‐selectin, Factor V, VII, VIII, PAI‐1) with dementia risk. Cox proportional hazards and conditional logistic regression models were used, adjusting for age, sex, education, cardiovascular risk factors, and ApoE‐ε4 genotype.

**Results:**

Higher platelet concentrations were associated with an increased risk of dementia (HR 1.24, 95% CI 0.99–1.55) as well as antiplatelet drug use (HR 1.46, 95% CI 1.02–2.09); Factor V was inversely associated with dementia risk (OR 0.48, 95% CI 0.28–0.83) and AD risk (OR 0.32, 95% CI 0.15–0.72), suggesting a neuroprotective effect whilst no significant associations were found for fibrinogen, *p*‐selectin, Factor VII, or VIII.

**Conclusion:**

Higher platelet levels and antiplatelet drug use are associated with increased dementia risk, while Factor V may be protective. These findings highlight the importance of coagulation biomarkers in early dementia detection and potential therapeutic strategies.